# Supplementary figures and images for: Blood multiomics reveal insights into population clusters with low prevalence of diabetes, dyslipidemia and hypertension
Source: PLoS One. 2020 Mar 5;15(3):e0229922. doi: 10.1371/journal.pone.0229922 (PMC7058291; doi:10.1371/journal.pone.0229922)

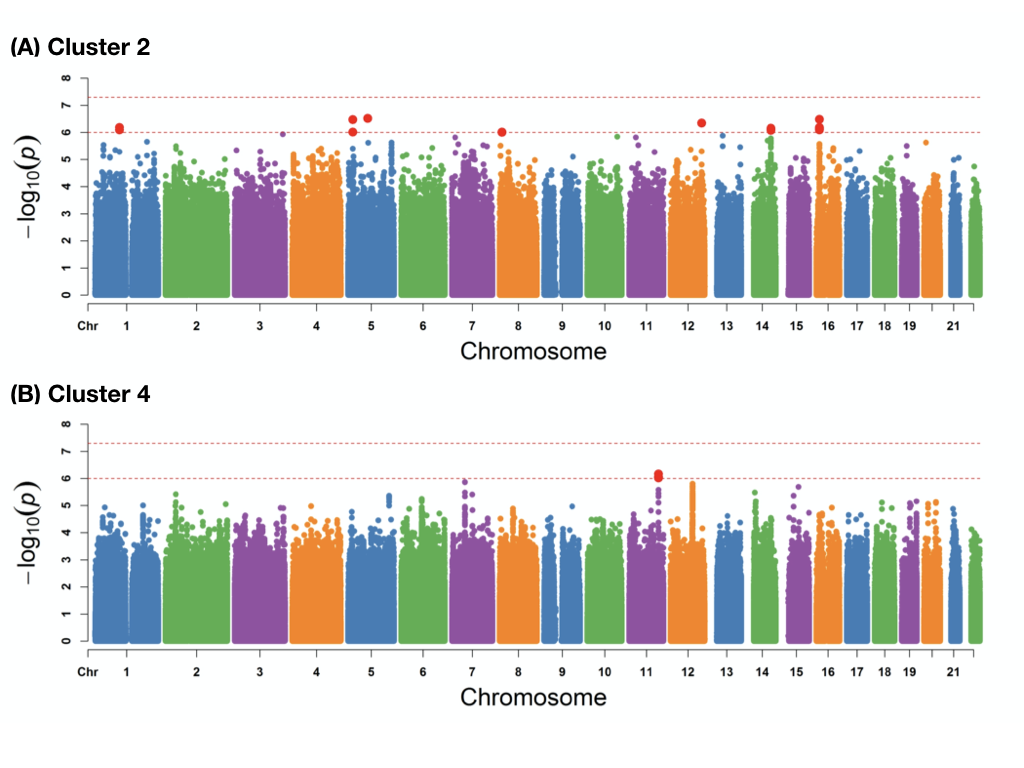

Supplement: S1 Fig — Red dots represent SNPs with p < 10−6. (TIF) [file pone.0229922.s001.tif]

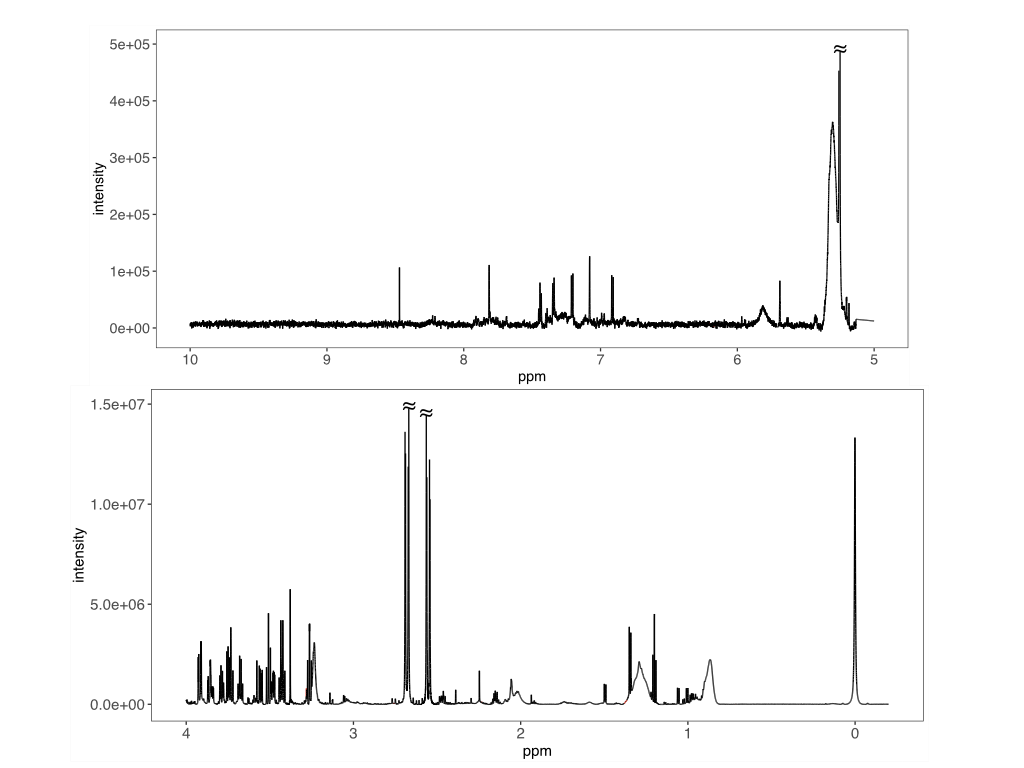

Supplement: S2 Fig — The water region (4–5 ppm) has been removed for clarity. (TIF) [file pone.0229922.s002.tif]

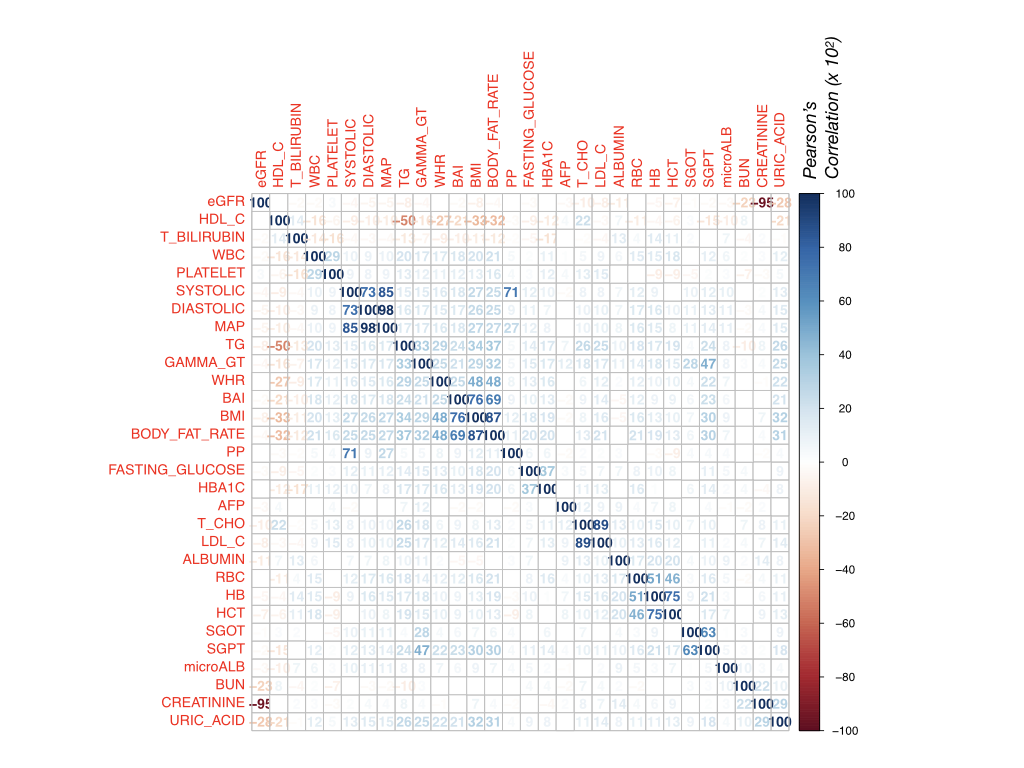

Supplement: S3 Fig — Blue and red colors indicate positive and negative correlations, respectively. Numbers denote the Pearson’s correlation between the traits. (TIF) [file pone.0229922.s003.tif]

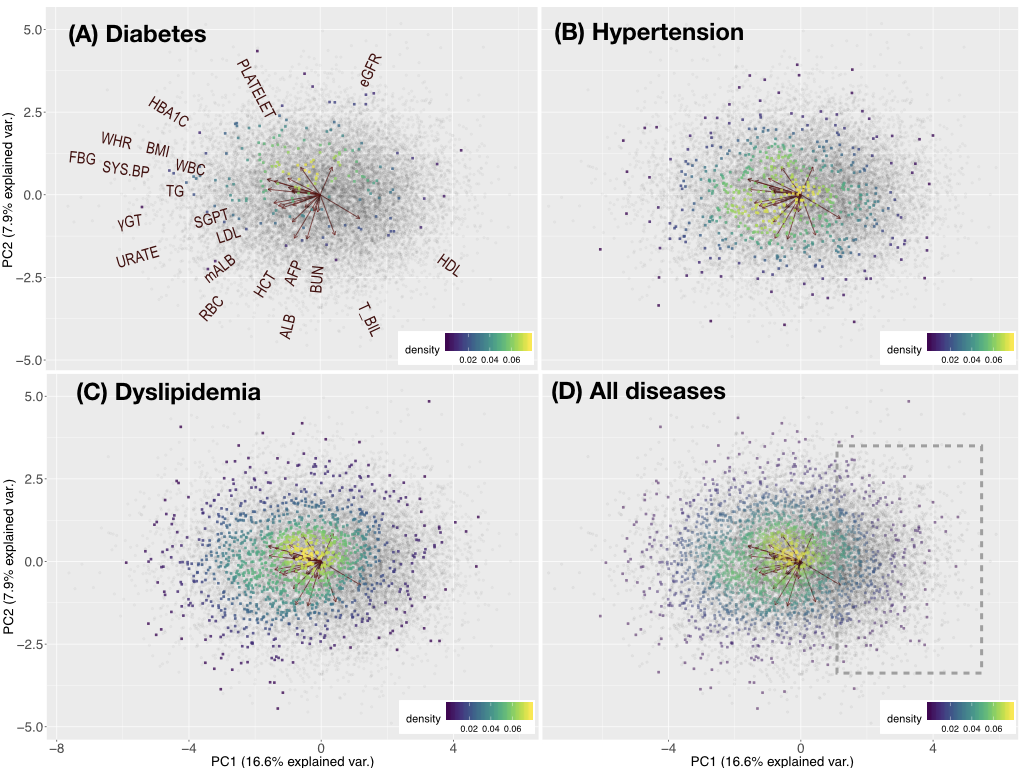

Supplement: S4 Fig — (TIF) [file pone.0229922.s004.tif]
