# Supplementary material for: Blood multiomics reveal insights into population clusters with low prevalence of diabetes, dyslipidemia and hypertension
Source: PLoS One. 2020 Mar 5;15(3):e0229922. doi: 10.1371/journal.pone.0229922 (PMC7058291; doi:10.1371/journal.pone.0229922)
Supplement: S1 Table — (DOCX) [file pone.0229922.s005.docx]

**S1 Table.** **SNPs associated with low disease prevalence.**

| Snp | Gene | Gene Function | p-value |
| --- | --- | --- | --- |
| rs651821 | *APOA5* | regulates the plasma triglyceride levels | 2.23E−16 |
| rs264 | *LPL* | lipoprotein lipase | 2.08E−06 |
| rs368971943 | *TRPC4* | acts as a cell-cell contact-dependent endothelial calcium entry channel | 9.38E−07 |
| rs71556736 | *MLXIPL* | forms a heterodimeric complex and binds and activates, in a glucose- dependent manner, carbohydrate response element (ChoRE) motifs in the promoters of triglyceride synthesis genes. | 3.00E−07 |
| rs10137529 | *HIF1A* | cellular response to systemic oxygen levels | 2.94E−06 |
| rs7301566 | *LIMA1* | encodes a cytoskeleton-associated protein that inhibits actin filament depolymerization | 4.38E−06 |
