# Supplementary material for: Blood multiomics reveal insights into population clusters with low prevalence of diabetes, dyslipidemia and hypertension
Source: PLoS One. 2020 Mar 5;15(3):e0229922. doi: 10.1371/journal.pone.0229922 (PMC7058291; doi:10.1371/journal.pone.0229922)
Supplement: S1 File — (DOCX) [file pone.0229922.s006.docx]

Blood multiomics reveal insights into population cluster with low prevalence of diabetes, dyslipidemia and hypertension

Ming-Wei Su^1^^¶^, Chung-ke Chang^1¶^, Chien-Wei Lin^1^, Shiu-Jie Ling^2^, Chia-Ni Hsiung^1,3^, Hou-Wei Chu^1^, Pei-Ei Wu^1^, Chen-Yang Shen^1,4*^

^1^Institute of Biomedical Sciences, Academia Sinica, Taipei, Taiwan

^2^Wego Private Bilingual Senior High School, Taipei, Taiwan

^3^Institute of Bioinformatics and Structural Biology, National Tsing Hua University, Hsinchu, Taiwan

^4^College of Public Health, China Medical University, Taichung, Taiwan

**^*^Corresponding author**

E-mail: [bmcys@ibms.sinica.edu.tw](mailto:bmcys@ibms.sinica.edu.tw) (C.-Y.S.)

^¶^M.-W. Su and C. Chang contributed equally to this work

**Supplemental Methods**

*Pre-processing of phenotypic cluster analysis data:* Missing values for the quantitative traits were imputed with the *k*-nearest neighbor algorithm. Values measured for each quantitative trait were stratified by age and sex; if the value distribution of the trait was highly skewed, we applied a log-transformation to force normality. Z-scoring or rank-based inverse normal transformation was then applied on the resulting residuals for normalization.

*k-means clustering:* The essence of the *k*-means clustering algorithm is to arrange study participants into phenotype-based clusters that maximize the feature distances between clusters and minimize the feature distances within clusters. The steps involved are listed below:

1. Randomly select *k* cluster centers.
2. Assign each data point to the nearest cluster center.
3. After all data points have been assigned, calculate the center for each cluster and reassign it as the new cluster center.
4. Repeat steps 2-3 until the cluster assignment of each data point becomes invariant or the maximum number of iterations (1,000) is reached.

*Preparation of blood plasma samples for NMR:* All chemicals were purchased from Merck (Darmstadt, Germany) and Cambridge Isotope Laboratories (Tewksbury, MA, USA). Blood plasma samples with sodium citrate as the anticoagulant were obtained from the Taiwan Biobank and stored at -80 °C. The samples were directly transferred from a -80 °C storage freezer to a 4 °C refrigerator on the day before the NMR experiment. The next morning, the samples were centrifuged at 14,000 rpm and 4 °C for 5 min in a Hitachi CT-15RE tabletop centrifuge equipped with a T15A61 rotor (Hitachi, Japan). A 110-μl aliquot of the supernatant was mixed with 220 μl of 1.5× NMR buffer consisted of 0.05 M Na_2_HPO_4_, 0.03% NaN_3_, 0.06 mM TSP-d_4_ (3-(trimethylsilyl)propionic-2,2,3,3-d_4_ and 15% ^2^H_2_O. A total of 320 μl of the buffered plasma solution was then transferred to a 4-mm (i.d.) NMR tube (Norell, US) and kept on ice until data acquisition.

*NMR experiments:* The ^1^H CPMG-PRESAT (Carr-Purcell-Meiboom-Gill with solvent presaturation) one-dimensional spectra of the blood plasma samples were acquired on a Bruker Avance 800-MHz spectrometer (Bruker, Rheinstetten, Germany) equipped with a 5-mm TCI probe at 25 °C using a spectral width of 20 ppm and 32,768 time domain points (corresponding to 16,384 complex points on the free-induction decay data). The inter-scan delay was set to 2 s and 128 transients were collected per spectrum. Each sample was left at room temperature (ca. 23 °C) for 7 min before being loaded onto the spectrometer. Another 5 min were allowed for temperature equilibration within the spectrometer. Standard operating procedures before each sample acquisition involved automatic tuning and matching of the proton channel, automatic shimming of the Z-coils, automatic three-dimensional shimming, another round of automatic shimming for the Z-coils, and finally automatic 90-degree pulse calibration.
