# Supplementary material for: Blood multiomics reveal insights into population clusters with low prevalence of diabetes, dyslipidemia and hypertension
Source: PLoS One. 2020 Mar 5;15(3):e0229922. doi: 10.1371/journal.pone.0229922 (PMC7058291; doi:10.1371/journal.pone.0229922)
Supplement: Attachment — Submitted filename: Response_to_reviewers.docx [file pone.0229922.s007.docx]

8 January, 2020

Dear Editor,

We thank you and both reviewers for pointing out oversights and providing valuable comments to our manuscript. We have secured the services of a professional English editor to help us revise the manuscript, and hopefully this revision will address most of the language issues. A point-by-point response to the review follows:

*Journal format requirements:*

1. *Please ensure that your manuscript meets PLOS ONE's style requirements, including those for file naming. The PLOS ONE style templates can be found at* [*http://www.plosone.org/attachments/PLOSOne_formatting_sample_main_body.pdf*](http://www.plosone.org/attachments/PLOSOne_formatting_sample_main_body.pdf) *and* [*http://www.plosone.org/attachments/PLOSOne_formatting_sample_title_authors_affiliations.pdf*](http://www.plosone.org/attachments/PLOSOne_formatting_sample_title_authors_affiliations.pdf)

Answer: We have made the appropriate changes according to the style templates.

2. *In ethics statement in the manuscript and in the online submission form, please provide additional information about the patient records used in your retrospective study. Specifically, please ensure that you have discussed whether all data were fully anonymized before you accessed them and/or whether the IRB or ethics committee waived the requirement for informed consent. If patients provided informed written consent to have data from their medical records used in research, please include this information.*

Answer: We did not utilize patient records from any medical institution, but rather relied on our subjects’ self-reported disease status, which is a standard multiple-choice question included in the Taiwan Biobank questionnaire. All the data were fully anonymized before access, and written informed consent from each and every Taiwan Biobank subject was given for the broad scientific use of the data. We have added the relevant parts to the first paragraph of the Materials and Methods section.

3. *We note that you have indicated that data from this study are available upon request. PLOS only allows data to be available upon request if there are legal or ethical restrictions on sharing data publicly. For more information on unacceptable data access restrictions, please see* [*http://journals.plos.org/plosone/s/data-availability#loc-unacceptable-data-access-restrictions*](http://journals.plos.org/plosone/s/data-availability#loc-unacceptable-data-access-restrictions)*. In your revised cover letter, please address the following prompts:*

*a) If there are ethical or legal restrictions on sharing a de-identified data set, please explain them in detail (e.g., data contain potentially sensitive information, data are owned by a third-party organization, etc.) and who has imposed them (e.g., an ethics committee). Please also provide contact information for a data access committee, ethics committee, or other institutional body to which data requests may be sent.*

*b) If there are no restrictions, please upload the minimal anonymized data set necessary to replicate your study findings as either Supporting Information files or to a stable, public repository and provide us with the relevant URLs, DOIs, or accession numbers. For a list of acceptable repositories, please see* [*http://journals.plos.org/plosone/s/data-availability#loc-recommended-repositories*](http://journals.plos.org/plosone/s/data-availability#loc-recommended-repositories)*.*

*We will update your Data Availability statement on your behalf to reflect the information you provide.*

Answer: Data from the Taiwan Biobank is subject to the “Biobank Examination Guidelines for the International Transmission of Biobank Data and the Export of Biospecimen Derivatives” (temporary translation) issued by the Ministry of Health and Welfare of Taiwan (<http://www.rootlaw.com.tw/LawArticle.aspx?LawID=A040170031038200-1060912> in Chinese). As such, access to biobank-derived data regardless of anonymization status, such as the ones used to conduct our study, by foreign entities requires explicit examination and permission by the Ministry. This includes the deposition of data to servers whose physical locations are outside of Taiwan. In order to be granted access, the party requesting the data may contact the authors or the Taiwan Biobank directly (biobank@gate.sinica.edu.tw), which may then submit an international data transfer application for review by the Ministry.

4. *Please include a copy of Table 2 which you refer to in your text on page 11.*

Answer: We apologize for the oversight. Table 2 has been included in the manuscript in the subsection titled “Common phenotypic traits define disease status in different clusters”.

5. *Please include captions for your Supporting Information files at the end of your manuscript, and update any in-text citations to match accordingly. Please see our Supporting Information guidelines for more information:* [*http://journals.plos.org/plosone/s/supporting-information*](http://journals.plos.org/plosone/s/supporting-information)*.*

Answer: The captions have been added and the in-text citations updated according to the guidelines.

*Response to Reviewer #1 comments:*

*This study aims to investigate the use of blood multiomics to reveal insights into population cluster with low prevalence of diabetes, dyslipidemia and hypertension. This is a very interesting work with well-organized format. I would recommend its acceptance after some minor corrections in English language.*

Answer: We have sent the manuscript to a professional English editor for English editing. The revised manuscript has incorporated all the recommended language changes.

*Response to Reviewer #2 comments:*

1. *line 146, "Table 2" is not attached.*

Answer: Attached in the revision. Again, we apologize for the oversight.

*2. line 168, "Table 1" is wrong and must be revised.*

Answer: We were referring to Fig 2 instead. Apologies for the oversight. We have also amended Fig 2 such that the connection between rs651821 and the APOA5 gene are better marked for the reader.

*3. I strongly suggested a native English writer to edit the whole manuscript.*

Answer: We have sent the manuscript to a professional English editor for English editing. The revised manuscript has incorporated all the recommended changes in language.

We hope that the revised manuscript and associated files is now fit for publication in *PLOS One*.

Sincerely yours,

Chen-Yang Shen, PhD

Institute of Biomedical Sciences

Academia Sinica

Taipei, Taiwan 115
